# Context‐specific anti‐inflammatory roles of type III interferon signaling in the lung in nonviral injuries

**DOI:** 10.14814/phy2.70104

**Published:** 2024-10-25

**Authors:** Jingjing Feng, Jooyoung Kim, Victoria D. Wang, De Chang, Hongbo Liu, William G. Bain, Keven M. Robinson, Zhijun Jie, Sergei V. Kotenko, Charles S. Dela Cruz, Lokesh Sharma

**Affiliations:** ^1^ Department of Pulmonary and Critical Care Medicine, Shanghai Fifth People's Hospital, Center of Community‐Based Health Research Fudan University Shanghai China; ^2^ Section of Pulmonary, Critical Care and Sleep Medicine Yale School of Medicine New Haven Connecticut USA; ^3^ Division of Pulmonary, Allergy, Critical Care, and Sleep Medicine, Department of Medicine University of Pittsburgh School of Medicine Pittsburgh Pennsylvania USA; ^4^ Department of Pulmonary and Critical Care Medicine at the Seventh Medical Center, College of Pulmonary and Critical Care Medicine of the Eighth Medical Center Chinese PLA General Hospital Beijing China; ^5^ Veterans Affairs Pittsburgh Healthcare System Pittsburgh Pennsylvania USA; ^6^ Department of Biochemistry and Molecular Biology Rutgers New Jersey Medical School New Brunswick New Jersey USA

**Keywords:** bleomycin, fibrosis, lung inflammation, lung injury, type III interferons

## Abstract

Type III interferons (λ1, λ2, and λ3) are potent antiviral cytokines in the lung. However, their roles in nonviral lung injuries are less well understood. This study investigates the activation of type III interferon signaling in three distinct models of lung injuries caused by diverse stimuli: the bacterial pathogen Pseudomonas aeruginosa, bacterial endotoxin LPS, and the chemotherapeutic agent bleomycin. Our data show that, despite inducing a potent inflammatory response, Pseudomonas and LPS did not increase IFNλ secretion. In contrast, bleomycin instillation increased secretion of IFNλ in the airways at both early and late time points. Consistent with limited secretion, type III interferon signaling had a minimal role in the host response to both Pseudomonas and LPS, as measured by pathogen burden, inflammatory response, and lung injury. Conversely, a deficiency in type III interferon signaling led to increased inflammatory signaling and elevated acute lung injury in the bleomycin model on day 3. This elevated early injury resulted in impaired recovery in IFNLR1 knockout mice, as evidenced by their recovery from bleomycin‐induced weight loss. Taken together, these data suggest a context‐specific activation of type III interferon signaling, where it plays an anti‐inflammatory role in the lung.

## 1 INTRODUCTION

Interferon lambdas (λ1, λ2, and λ3), collectively known as type III interferon, are a crucial component of the innate immune response against viral pathogens at mucosal barriers, including the respiratory system (Kotenko et al., 2003; Kotenko & Durbin, 2017). Upon detection of viral pathogens, various cells, including epithelial cells and immune cells, produce interferon λ (Syedbasha & Egli, 2017). This cytokine initiates an immune response through the heterodimeric interferon λ receptor (IFNLR) complex, which consists of the α‐subunit (IL‐28RA or IFNLR1) and β‐subunit (IL‐10RB), inducing the activation of Janus kinase‐1, tyrosine kinase‐2, and phosphorylation of STAT‐1 and 2, leading to the induction of interferon‐stimulated genes (ISGs) similar to type I interferon (Bender et al., 2015; Hamming et al., 2013; Pervolaraki et al., 2018). Compared to the type I interferon receptor (IFNAR1/2) and IL‐10Rβ, IFNLR1 is mostly expressed on the epithelial cells of the lung, liver, and gut. Many studies have shown that interferon λ plays a vital role in limiting viral replication and spread within the host, thereby controlling viral infections such as influenza, respiratory syncytial virus (RSV), and SARS‐CoV‐2 (Crotta et al., 2013). Additionally, interferon λ has been implicated in modulating the immune response to bacterial pathogens, such as *Staphylococcus aureus*, *Pseudomonas aeruginosa*, and *Mycobacterium tuberculosis*, contributed to bacterial clearance and resolution of infection, particularly in the lung epithelial cells (Cohen & Prince, 2013; Planet et al., 2016; Travar et al., 2014). In contrast, therapeutic IFN λ impairs bacterial clearance during influenza superinfection by reducing neutrophil uptake of MRSA and *S*. *pneumoniae* in the infected lung (Rich et al., 2019).

Despite extensive research into the role of interferon λ in infectious diseases, its impact on the inflammatory response in the lung in noninfectious pathologies have received limited attention. In this study, we aimed to address the function of interferon λ in the context of lung injury induced by bacterial and noninfectious insults. We utilized three distinct models of lung injury, employing *Pseudomonas aeruginosa* (PA01 strain) infection, lipopolysaccharide (LPS) administration, and bleomycin‐induced lung injury model, to investigate the role of type III interferon signaling in inflammatory response and lung injury. Through genetic knockout mouse models, we demonstrated that absence of type III interferon signaling had limited effect on the inflammatory responses in bacterial or LPS injury models but has anti‐inflammatory effects during bleomycin‐induced lung injury.

## 2 MATERIALS AND METHODS

### 2.1 Mouse models

All animal experiments performed in this study were approved by IACUC at the Yale University. Animals were housed in standard 12‐h day night cycle and provided standard rodent chow and water ad‐libitum. Both male and female mice (age 8–14 weeks) were used in these experiments with similar findings. Each experiment had *N* = 3–6 depending upon the size of the litter. Wild‐type mice were obtained from Jackson Laboratory and IFNLR1KO mice were provided by Sergei Kotenko as described before (Sharma et al., 2022). For the Pseudomonas infection model and bleomycin‐induced fibrosis, mice were intratracheally administered with 2.5 × 10^6^ CFUs of PA01 and 1.5 U/Kg of bleomycin as previously described (Peng et al., 2022). For the LPS model, mice received 5 μg of E. coli LPS. Mice were monitored every day, weight changes were measured every other day, and harvested BAL and lung tissues on indicated days. Animals were euthanized using intraperitoneal injection of urethane followed by removal of vital organs such as lung to ensure euthanasia.

### 2.2 Bronchoalveolar lavage fluids, bacterial and cell counts

Bronchoalveolar lavage (BAL) fluids were obtained from mice by instilling two aliquots of 0.75 mL of sterile PBS. BAL was plated for enumerating bacteria along with left lung homogenized in 1 mL of sterile PBS on minimal agar on Vogel's media as described before (Sharma et al., 2018). BAL was then centrifuged to separate cells from the supernatant. Cells were resuspended in 300 μL of phosphate buffered saline (PBS) and used to count white blood cells are and red blood cells using Coulter counter. Cell free supernatants were frozen at −80°C until further measurements of cytokines or total protein content were performed.

### 2.3 Total protein

Total protein in the BAL was assessed as a marker of lung permeability using Bicinchoninic acid (BCA) kit from Pierce (Catalog # 23225).

### 2.4 ELISA of BAL

ELISA assays were performed using Duoset kits from R&D as per instructions provided by the manufacturer. Following cytokines were measured: IFN‐λ (Cat# DY1789B), CXCL1 (KC, Cat# DY453), IL‐1β (Cat# DY401), TNFα (Cat# DY410), and IL‐6 (Cat# DY406).

### 2.5 Collagen measurements

Collagen levels were measured using Sircol assay kit from Biocolor Life Science assays (Cat #S1111). Histological analysis of collagen deposition was performed using 10 μm thick lung section stained with trichrome staining using kit from Abcam (cat# ab150686).

### 2.6 Statistics

We conducted at least two experiments for each set to confirm reproducibility. Two variables were compared using Student's *t*‐test, and multiple comparisons were analyzed using two‐way ANOVA. A *p* value of <0.05 was considered statistically significant. RAW data will be available to anyone with a reasonable request with the corresponding author.

## 3 RESULTS

### 3.1 Upregulation of type III interferon by different stimuli

**FIGURE 1:**
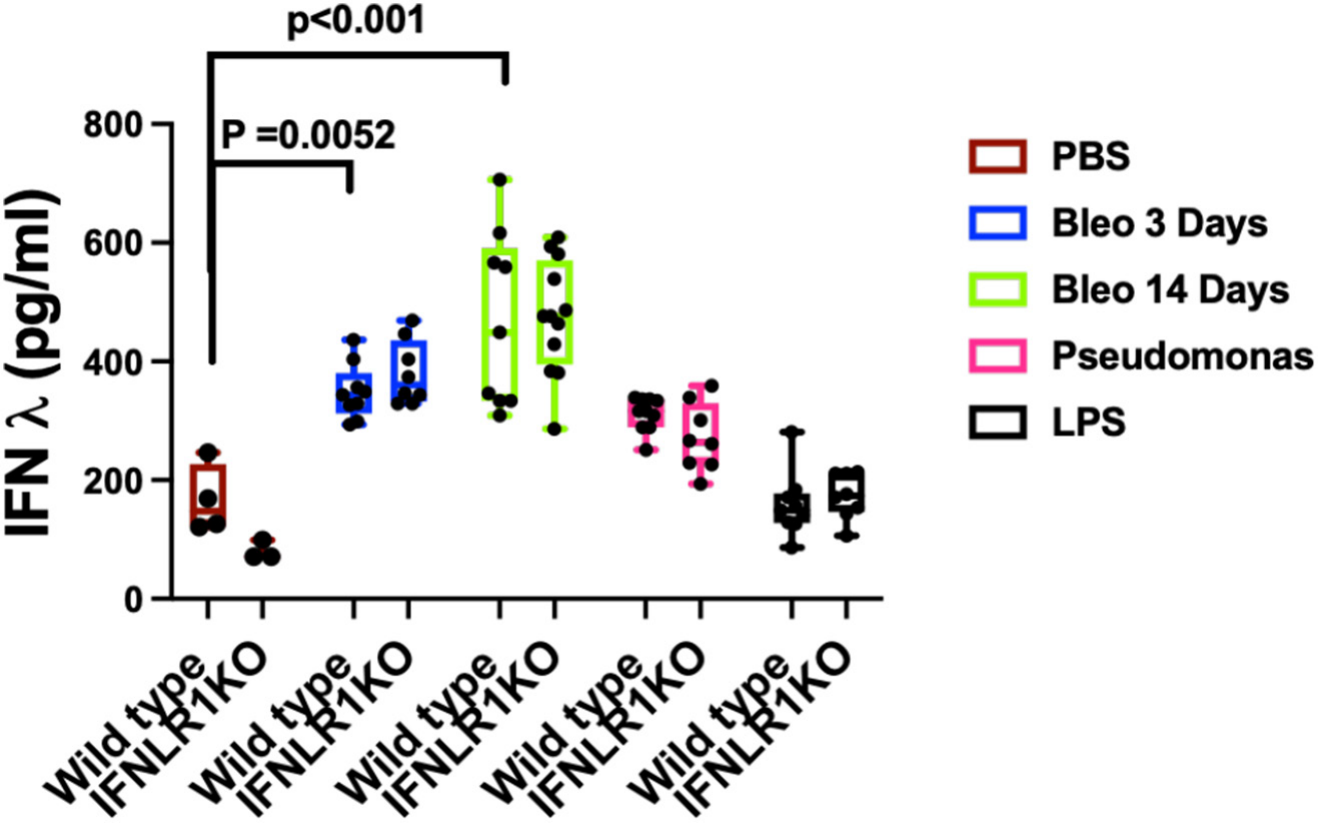
Stimuli specific elevation of type III interferons in the lung: BAL samples were obtained from wild type (C57/B6) and co‐housed age‐ and sex‐matched IFNLR1 KO mice that were either intratracheally infected with *Pseudomonas aeruginosa* (12 h) or administered with LPS (12 h) or bleomycin (3 or 14 days). BAL samples from mock (PBS) administered mice were obtained as controls. Type III interferon levels were measured by ELISA assays. Data are obtained from two independent experiments performed for each stimulus. Statistically significant *p* values are indicated using two‐way ANOVA and Sidak's multiple comparisons test. *N* = 4 wild type, 3 IFNLR1 KO for PBS, *N* = 9 wild type, 8 IFNLR1KO for bleomycin 3 days, *N* = 9 wild type, 12 IFNLR1KO for bleomycin 14 days, *N* = 10 wild type, 8 IFNLR1KO for Pseudomonas, *N* = 9 wild type, 8 IFNLR1KO for LPS. The data are plotted as box that shows 25th and 75th percentile and the middle line indicating median and whiskers that show spread of the data. This is consistent throughout all the figures.

We first investigated the regulation of type III interferon response by measuring the levels of IL‐28A/B (lFNλ2 and IFNλ3) in three different stimuli in mouse models. These stimuli included bacterial pathogen *Pseudomonas aeruginosa* (PA), bacterial lipopolysaccharide (LPS), and chemotherapeutic agent bleomycin, which causes acute lung injury during early phase (2–4 days), resolution of which leads to a fibrotic response (in 2–3 weeks). We measured the levels of IL‐28A/B in the BAL samples of mice infected with PA, or intratracheal LPS for 12 h or intratracheal bleomycin for 3 days (acute lung injury phase) and 14 days (fibrotic phase). Our data show that while bleomycin induced a strong interferon λ response at both 3 day and 14 days post infection (Figure 1). In contrast, infection with PA or instillation of LPS did not induce a significant upregulation of type III interferon response. Interestingly, we did not see any differences in the levels of type III interferons between wild type and IFNLR1 KO mice, indicating that interferon signaling has limited role on its own secretion.

### 3.2 Role of type III interferons in *Pseudomonas aeruginosa* infection

**FIGURE 2:**
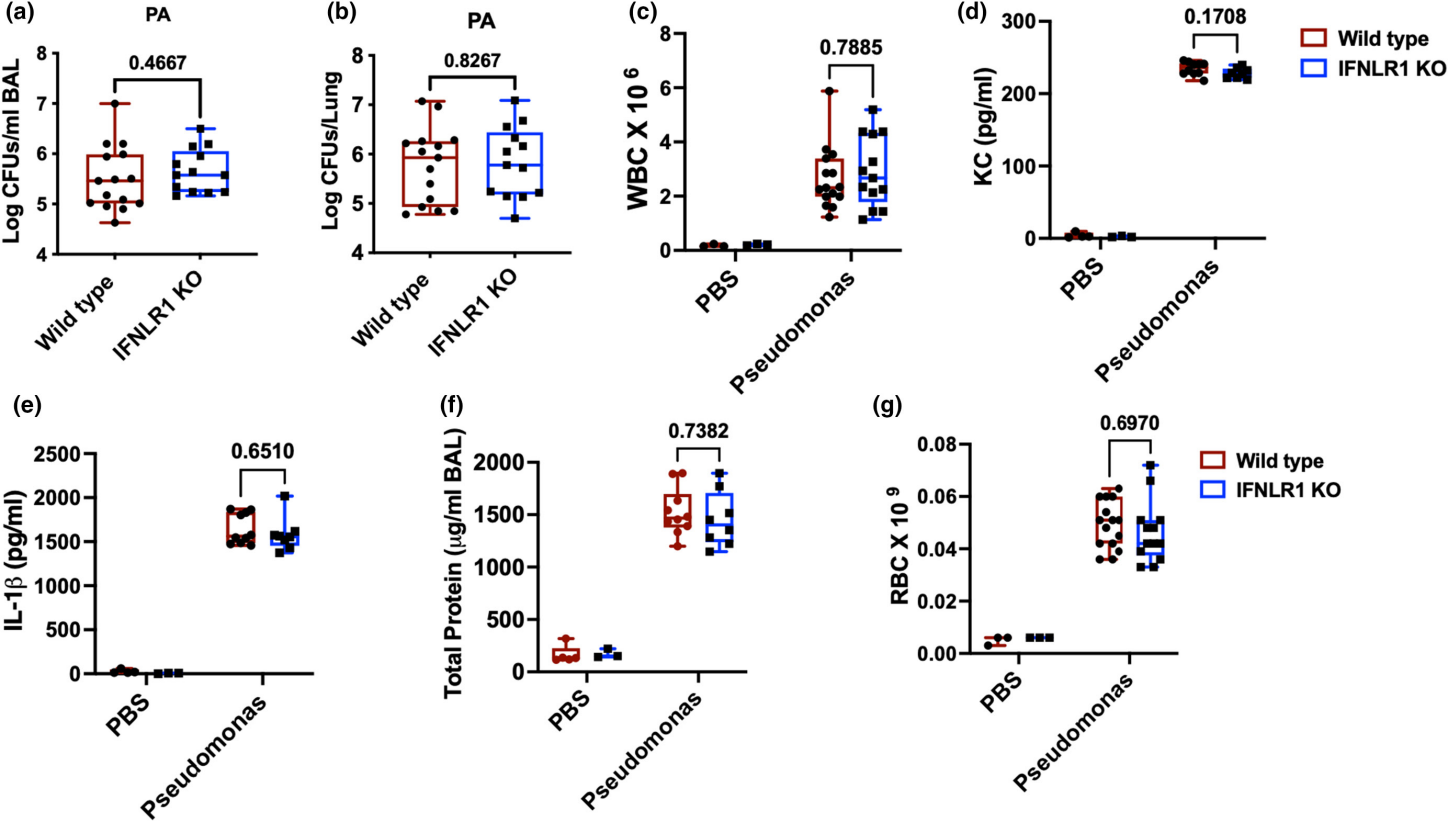
A minimal role of type III interferon signaling in *Pseudomonas aeruginosa* infection: Wild type and IFNLR1 KO mice were infected with 2.5 × 10^6^ CFUs of PA01 strain of *Pseudomonas aeruginosa* (PA) for 12 h. Serial dilutions of BAL samples (a) and homogenized left lung (b) were plated on minimal agar to enumerate the bacterial numbers. WBCs were counted using Coulter counter (c). ELISA assays were performed on the BAL samples of Pseudomonas or mock infected mice using Duoset ELISA kits for KC and IL‐1β (d and e). Total protein levels were measured by BCA assay (f). Red blood cells were counted by Coulter counter (g). Hereafter, in all figures, wild type mice are indicated by solid circles in red boxes and IFNLR1 KO mice are indicated in solid squares in blue boxes. Data are pooled from three independent experiments (*N* = 15 wild type and 13 IFNLR1 KO) except for total protein (f) and cytokines, which are from pooled from two independent experiments (*N* = 10 wild type and 8 INFLR1 KO). *p* values are indicated using unpaired *t*‐test or two‐way ANOVA and Sidak's multiple comparisons test. For baseline samples, *N* = 3 for each genotype in (c) and (g), *N* = 4 (e), or 5 (f) wild type and 3 INFLR1 KO for PBS samples. *p* values are indicated on top of the graph.

Next, we investigated the role of type III interferon signaling in host response to bacterial infection using Gram negative opportunistic pathogen *Pseudomonas aeruginosa*. We utilized wild type and IFNLR1 deficient (KO) mice. Mice inoculated with PA were euthanized at 12 h to measure bacterial burden and lung injury. Our data show that absence of type III interferon signaling did not change the overall bacterial burden as measured in both bronchoalveolar lavage fluid (BAL) (Figure 2a) and lung tissue (Figure 2b). Pseudomonas infection increased significant leukocyte infiltration in the airways as compared to the mock infected mice (Figure 2c). However, in line with minimal secretion of type III interferon in PA infection, we did not observe any difference in recruitment of immune cells in the lung during PA infection between two genotypes (Figure 2c). Consistent with unaltered recruitment of immune cells, we observed similar levels of potent neutrophil chemokine KC between wild type and IFNLR1 KO mice (Figure 2d). Similarly, we did not observe any differences in IL‐1β levels among the infected mice between wild type and IFNLR1 KO mice (Figure 2e). Further, we investigated the effect of IFNLR1 on lung injury by measuring the presence of total protein content and red blood cells in the BAL fluid. Our data show that both wild type and IFNLR1 KO mice had significantly elevated levels of protein content compared to mock infected animals, but had no differences based on genotype (Figure 2f). Similar observations were made for RBC counts in the blood, that were not different between wild type and IFNLR1 KO mice (2G). These data suggest that there is a minimal role of type III interferon signaling in mediating bacterial clearance, inflammatory response, and lung injury during PA infections with PA01 strain.

### 3.3 Role of type III interferon in lipopolysaccharide‐induced acute lung injury

**FIGURE 3:**
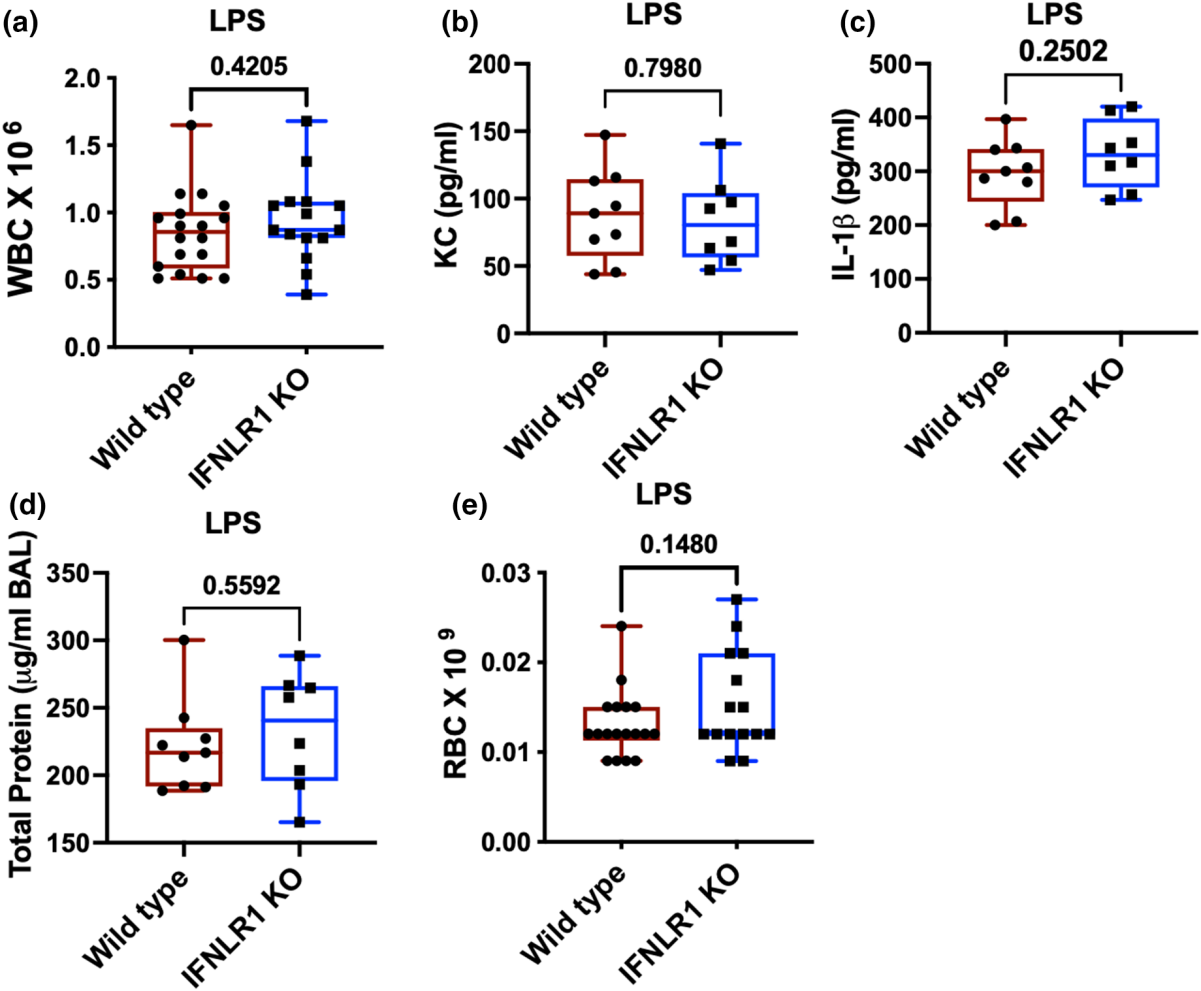
Dispensable role of type III interferon signaling in LPS model of lung injury: BAL samples were obtained from wild type and IFNLR1 KO mice that were intratracheally administered 5 μg of *E. coli* LPS for 12 h. Levels of total WBC were measured in the BAL (a) and the level of cytokines were measured in cell free BAL for KC and IL‐1β (b and c). Total protein content in the cell free BAL (d) and RBCs were measured (e). *N* = 18 wild type and 15 IFNLR1 KO mice for (a) and (e) pooled from four different experiments, *N* = 9 wild type and 8 IFNLR1 KO mice pooled from two independent experiments for (b), (c), and (d). *p* values are indicating on the graph using student's *t*‐test.

Given the complexity of bacterial infections, where inflammatory response changes based on the bacterial burden, we next aimed to investigate role of type III interferon signaling in LPS model. LPS causes inflammatory lung injury through TLR4 signaling. Our data show that there was similar inflammatory response in the lung based on the inflammatory cell recruitment in the lung (Figure 3a). Further, similar levels of inflammatory cytokines such as neutrophil chemokine KC (Figure 3b) and inflammasome cytokine IL‐1β (Figure 3c) were observed. Lung injury markers including total protein content (Figure 3d) and RBCs (Figure 3e) in the BAL were not different between wild type and IFNLR1 KO mice. These data suggest that type III interferon signaling has minimal role in controlling inflammatory and injury response in the lung during LPS simulation.

### 3.4 Role of type III interferon signaling in bleomycin‐induced acute lung injury

**FIGURE 4:**
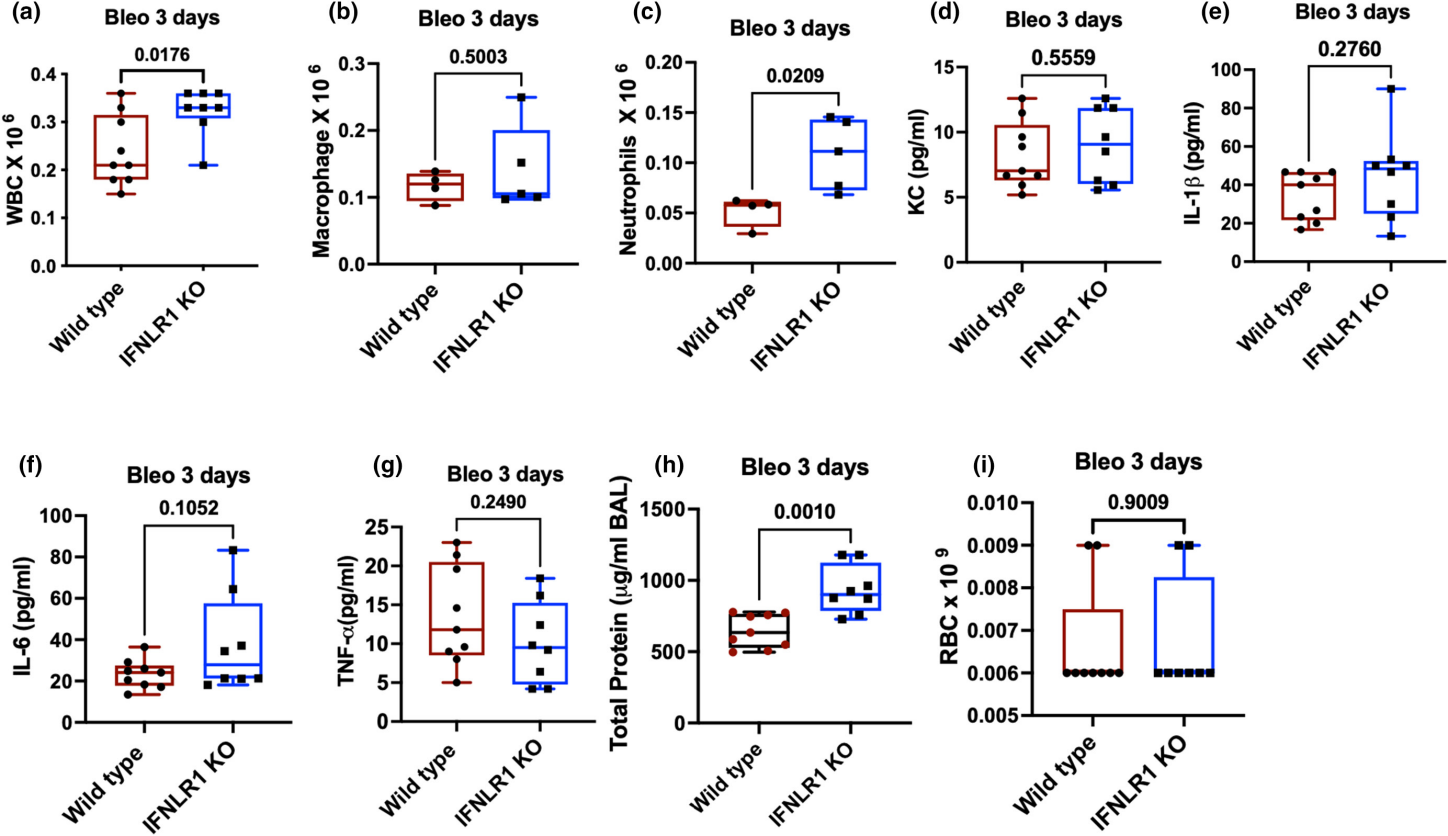
Anti‐inflammatory role of type III interferon signaling in bleomycin‐induced acute lung injury: Wild type and IFNLR1 KO mice were intratracheally administered 1.5 U/kg bleomycin by intratracheal route and euthanized on day 3 post infection. WBC counts (a), macrophages (b), and neutrophils (c), cytokines KC (d), IL‐1β (e), IL‐I (f), and TNFα (g). Total protein content (h) and red blood cell counts (i) in the BAL as markers of lung injury. *N* = 9 wild type and 8 IFNLR1 KO mice for all the figures from two independent experiments except for (b) and (c) where *n* = 4 wild type and 5 INFLR1 KO mice. Data are analyzed using *t*‐test and *p* values are indicated on top of the graph.

After establishing a minimal role of type III interferon signaling in bacterial infection and LPS‐induced lung injury, we investigated its role in bleomycin‐induced lung injury. It is important to note that unlike Pseudomonas infection and LPS stimulation, bleomycin administration led to significant elevation of interferon λ (Figure 1). We first investigated role of interferon λ on the acute lung injury caused by bleomycin on day 3. Our data show an elevation of immune cell infiltration in the airways as measured by BAL cell counts on day 3 in IFNLR1 KO mice compared to the wild‐type mice (Figure 4a). Our cell differential data show that this difference was not mediated by macrophages (Figure 4b) but can be largely attributed to elevated neutrophil counts in IFNLR1 KO mice (Figure 4c). We did not observe any differences between inflammatory cytokines such as KC, IL‐1β, IL‐6, or TNFα at this time point (Figure 4d–g). Of note, the levels of these cytokines, such as KC and IL‐1β were significantly low in the bleomycin model compared to LPS or bacterial infection models (Figures 2 and 3). In agreement with the increased inflammatory cell recruitment at this time, we observed a significantly elevated total protein content in the BAL of these mice (Figure 4h). However, we did not see any differences in the RBC counts in the BAL (Figure 4i). Taken together, these data suggest that type III interferon signaling has anti‐inflammatory and anti‐injury roles during acute lung injury caused by bleomycin.

### 3.5 Role of type III interferon signaling in bleomycin‐induced chronic lung injury

**FIGURE 5:**
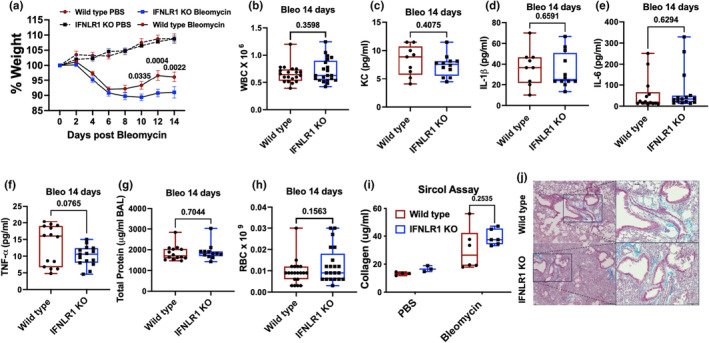
Role of type III interferon signaling in bleomycin‐induced chronic lung injury, fibrosis, and recovery: Wild type and IFNLR1 KO mice were administered 1.5 U/kg bleomycin or PBS by intratracheal route and followed up to 14 days with their body weight measured every other day (a). WBC (b), levels of KC (c), IL‐1β (d), IL‐6 (e), and TNFα (f) were measured to estimate inflammatory responses. Total protein in the BAL (g) and levels of RBCs in the BAL (h) were measured for lung injury. Collagen levels were measured on day 14 post infection in the left lung (i) and by trichrome staining of lung tissue (j). Data are pooled from at least two experiments for each measurement except collagen measurement, which was performed on one of the representative experiments. *p* values are indicated on the top of the graph using two‐way ANOVA with Tukey's multiple comparisons for (a), student's *t*‐test for (b)–(h), and two‐way ANOVA with Sidak's multiple comparison test for (i). For figure (a), *N* = 5 in PBS group for both genotypes, *N* = 16 wild type and 13 IFNLR1 KO mice from three independent experiments. *N* = 20 wild type and 21 IFNL1 KO mice from four independent experiments for (b) and (h). Cytokines were measured in two independent experiments *N* = 9 wild type and 11 IFNLR1 KO mice as shown in (c)–(f). *N* = 14 wild type and 12 INFLR1 KO from three independent experiments for (g). *N* = 4 wild type and 3 IFNLR1 KO mice for PBS and *N* = 6 wild type and 6 IFNLR1 KO each for bleomycin group in (i).

Having established a role of type III interferon signaling in regulating acute lung injury and inflammation in bleomycin model, we sought to determine its chronic consequences. Bleomycin leads to persistent lung inflammation and injury leading to weight loss and fibrosis. We followed these mice for 14 days to measure their body weight. Our data show that IFNLR1 KO mice had impaired recovery from the bleomycin‐induced weight loss compared to the wild‐type mice (Figure 5a). The inflammatory response on day 14, measured as total WBC counts in the BAL sample were not different (Figure 5b). At this time, we did not observe any differences in the cytokines such as KC, IL‐1β, IL‐6, or TNFα, between the two genotypes but were comparatively low (Figure 5c–f). Again, these cytokines were not dramatically induced in this model compared to the PA and LPS models (Figures 2 and 3). To determine the lung injury at this time point, we measured total protein content and RBC counts, both of which were similar between two genotypes (Figure 5g,h). Finally, to determine whether IFNLR1 deficiency had any effect on the fibrotic response, we measured collagen content in the lung to show that while collagen content increased after bleomycin‐induced lung injury, there was no statistically significant difference between genotypes (Figure 5i). These data are supported by histological analysis of fibrosis through trichrome staining, which shows presence of fibrosis in the lung but no differences between the two genotypes (Figure 5j). These data suggest that an impaired recovery from the weight loss might be due to initial elevated acute lung injury and/or slower injury repair in IFNLR1 KO mice. It is possible that effect of bleomycin on other organ system or direct effect on circulating cells may contribute to some of these effects; however, it is unlikely given intratracheal administration of the bleomycin in our model.

## 4 DISCUSSION

Interferons are cytokines with the potent ability to exert antiviral response against a wide range of viral pathogens (Galani et al., 2017; Jewell et al., 2010; Kotenko et al., 2003). Type III interferon, signaling has been shown to play a key role in viral infections such as influenza (Galani et al., 2017), metapneumovirus (Baños‐Lara et al., 2015), and coronavirus (Sharma et al., 2022), among many other viruses. However, limited studies have been conducted in investigating their role in regulating inflammatory response in the lung during nonviral and noninfectious insults. In this study, we investigated role of type III interferon signaling against three nonviral insults in the lung. Our data find a minimal role of type III interferons in bacterial infection and LPS models but demonstrate a role in the bleomycin model. This is in stark contrast with the type I interferon signaling, where absence of type I interferon signaling led to exacerbated inflammatory responses regardless of type of stimuli (Feng et al., 2024). Thus, our study establishes a context‐specific role of type III interferon signaling in the lung that is distinct from type I interferons. However, similar to type I interferon signaling, we expect a nuanced anti‐inflammatory role for type III interferon signaling. For example, supplementation of specific interferons, such as IFNα, led to a pro‐inflammatory and pro‐fibrotic response in the lung (Berkman et al., 1997, 2001), which was not recapitulated by IFNα A/D (Berkman et al., 2001). In contrast, hamsters injected with Poly I:C, a potent interferon inducer, had significantly ameliorated inflammatory and fibrotic responses (Giri & Hyde, 1988). This complexity may arise from differences in the effects of specific interferons, with 13 subtypes of interferon‐α, along with the impact of exogenous supplementation versus intracellular signaling in specific cells. Similar complexities are also expected to exist with type III interferons, where at least two subtypes, IFNλ2 and IFNλ3, present in mice, may exert differential activities depending on whether they are exogenously supplemented or produced intracellularly. More research is warranted to tease apart these complexities to establish therapeutically applicable roles of type III interferons in the lung.

Our data show that bacterial infection as well as stimulation with bacterial endotoxin did not stimulate much type III interferon response in vivo, compared to mock infected mice. A prior study has shown similar outcomes where infection with Pseudomonas led to only a transient increase in the interferon λ levels at 4 h and there were no differences at 18 h post infection (Cohen & Prince, 2013). These findings are supported by the fact that *Pseudomonas aeruginosa* can actively degrade interferon λ through its proteases (Sörensen et al., 2020). Prior studies into the role of interferon lambda signaling in bacterial infections provided conflicting data. In a prior study, deficiency of IFNLR1 receptor led to improved bacterial clearance in Pseudomonas model, which was not mimicked by our experimental approaches (Cohen & Prince, 2013). The possible differences in these studies maybe attributed to different strains of *Pseudomonas aeruginosa* (PAK vs. PA01) and timing (18 vs. 12 h post infection). However, we observed similar inflammatory cell recruitments, as demonstrated by this study between wild type and IFNLR1 KO mice post infection with Pseudomonas, along with no upregulation of interferon levels beyond 4 h, as reported in this study (Cohen & Prince, 2013). In Gram +ve bacterial infections interferon λ inhibited bacterial uptake in neutrophils and impaired bacterial clearance in a mouse model of post influenza bacterial infections with Gram +ve bacteria (Planet et al., 2016; Rich et al., 2019). In contrast, another study demonstrated that treatment of interferon λ2 ameliorated neutrophil mediated inflammatory response and improved clinical outcomes without improving bacterial clearance in Pseudomonas infection (Broquet et al., 2020). It is interesting to note that in the same study, neutralization of IFNλ2 did not alter clinical outcomes (Broquet et al., 2020). These data suggest that interferon λ may have limited and context‐specific roles in lung inflammation unless its induced either by supplementation (Broquet et al., 2020) or thorough a prior viral infection (Rich et al., 2019). These data are in agreement with a recent study, which found no role of interferon λ3 on MRSA infection in the lung (Rich et al., 2021).

In contrast, exposure to bleomycin, which causes epithelial injury (Reinert et al., 2013) led to a robust stimulation of type III interferon response at both early and later stage of injury (Figure 1). We observed small but significant elevation of inflammatory and injury response in the IFNLR1 KO mice during bleomycin model at early time points (day 3) (Figure 4). However, this difference in the inflammatory and injury response did not carry at the later time point of 14 days, where we observed no difference in inflammatory, injury, and fibrotic responses between wild type and IFNLR1 KO mice. However, despite similar injury response on day 14, IFNLR1 KO mice had impaired recovery from the bleomycin‐induced weight loss. Our data suggest that unlike type I interferon response, which has potent and wide ranging anti‐inflammatory properties in the lung (Feng et al., 2024), type III interferon signaling has a very mild and context‐specific anti‐inflammatory role in the lung. Another, key finding of our study is the lack of differences in type III interferon levels between wild type and IFNLR1 KO mice, which indicate a lack of feedback loop in type III interferon signaling in the lung. Prior studies have shown that mice that lack type I interferon receptor can also mount robust type III interferon response (Jewell et al., 2010). The precise mechanisms of interferon stimulation and role of its signaling in promoting its own secretion in vivo during lung injury remains to be full elucidated.

Functionally, type III interferons have significant overlap with type I interferon response including a large number of genes that are stimulated by both interferons. The functional differences between these two interferons arise from the location of their receptors (Kotenko & Durbin, 2017) and relative timing of secretion during an infection as well as some differences in the downstream genes (Pervolaraki et al., 2018). Type III interferons are believed to be the first response to a viral infection and aims to limit viral replication without eliciting an inflammatory response (Galani et al., 2017), which can be detrimental to the host by causing collateral damage to the tissue, as seen for type I interferons during viral infections (Davidson et al., 2014). The receptor expression of type III interferons is largely located on epithelial cells, although their presence and functions on immune cells have been reported (Galani et al., 2017; Kotenko & Durbin, 2017). This led to the belief that type III interferon response leads to silent clearance of the viral infection (Andreakos et al., 2019). This is in contrast to type I interferon, which are deemed inflammatory, and often associated with worse clinical outcomes, including in viral infections (Davidson et al., 2014). However, recent investigations have demonstrated detrimental effects of type III interferons in the lung where they impair resolution of tissue injury post lung infection with influenza by impairing epithelial regeneration (Major et al., 2020), a phenotype that was shared with type I interferons. Similar inhibitory effects of interferon λ has been reported for CD4 generation during RSV infection, which is also shared by type I interferons (Chi et al., 2006).

Despite type I interferon being correlated with higher inflammation and worse clinical outcomes in many diseases, experimental evidence suggests their anti‐inflammatory properties. One of the first experimental evidence was derived from mouse models of carrageenan induced foot pad swelling (Koltai & Mecs, 1973), which became well established in inflammatory diseases such as multiple sclerosis (Wee Yong et al., 1998). Our recent study demonstrated a conserved role for type I interferon in lung regardless of type of stimuli (Feng et al., 2024). Lack of effect in PA or LPS maybe partly attributed to limited secretion of type III interferons during pulmonary stimulation with bacteria or bacterial LPS or active degradation of interferon λ (Peignier & Parker, 2020; Sörensen et al., 2020). In contrast, these stimuli lead to robust activation and release of type I interferons (Yang et al., 2022). The absence of a robust release or accumulation of type III interferons might dampen its overall impact during these pathologies. On the contrary, robust induction in models such as bleomycin reveals their beneficial effects during lung inflammation in absence of a viral or bacterial pathogen. In conclusion, this study demonstrates that type III interferon signaling has anti‐inflammatory effects in certain contexts such as bleomycin‐induced lung injury. Their protective anti‐inflammatory effects are associated with the ability of the insult to induce the secretion of type III interferons.

## FUNDING INFORMATION

Lokesh Sharma is supported by Francis Family Foundation and American Lung Association Catalyst Award. Charles S Dela Cruz is supported by U.S. Department of Defense and U.S. Department of Veterans Affairs.

## ETHICS STATEMENT

All experimental procedures were approved by the Yale Institutional Animal Care and Use Committee (IACUC). The study adhered to ethical standard set by IACUC.

## Data Availability

All the raw data are available to the corresponding author (LS) and can be obtained after a reasonable request.
